# Evaluation of Cancer Deaths Attributable to Tobacco in California, 2014-2019

**DOI:** 10.1001/jamanetworkopen.2022.46651

**Published:** 2022-12-14

**Authors:** Frances B. Maguire, Ani S. Movsisyan, Cyllene R. Morris, Arti Parikh-Patel, Theresa H. M. Keegan, Elisa K. Tong

**Affiliations:** 1 California Cancer Reporting and Epidemiologic Surveillance Program, University of California Davis Comprehensive Cancer Center, Sacramento; 2 Center for Oncology Hematology Outcomes Research and Training, Division of Hematology and Oncology, University of California Davis School of Medicine, Sacramento; 3 Department of Internal Medicine, University of California Davis, Sacramento

## Abstract

**Importance:**

California’s tobacco control efforts have been associated with a decrease in cancer mortality, but these estimates are based on smoking prevalence of the general population. Patient-level tobacco use information allows for more precise estimates of the proportion of cancer deaths attributable to smoking.

**Objective:**

To calculate the proportion (smoking-attributable fraction) and number (smoking-attributable cancer mortality) of cancer deaths attributable to tobacco use using patient-level data.

**Design, Setting, and Participants:**

The smoking-attributable fraction and smoking-attributable cancer mortality were calculated for a retrospective cohort of patients whose cancer was diagnosed from 2014 to 2019 with at least 1 year of follow-up using relative risks from large US prospective studies and patient-level smoking information. Follow-up continued through April 2022. A population-based cohort was identified from the California Cancer Registry. Participants included adults aged 20 years and older with a diagnosis of 1 of the 12 tobacco-related cancers (oral cavity or pharynx, larynx, esophagus, lung, liver, stomach, pancreas, kidney, bladder, colon or rectum, cervix, and acute myeloid leukemia).

**Exposures:**

Tobacco use defined as current, former, or never.

**Main Outcomes and Measures:**

The primary outcomes were the smoking-attributable fraction and smoking-attributable cancer mortality for each of the 12 tobacco-related cancers over 2 time periods (2014-2016 vs 2017-2019) and by sex.

**Results:**

Among 395 459 patients with a tobacco-related cancer, most (285 768 patients [72.3%]) were older than 60 years, the majority (228 054 patients [57.7%]) were non-Hispanic White, 229 188 patients were men (58.0%), and nearly one-half (184 415 patients [46.6%]) had lung or colorectal cancers. Nearly one-half of the deaths (93 764 patients [45.8%]) in the cohort were attributable to tobacco. More than one-half (227 660 patients [57.6%]) of patients had ever used tobacco, and 69 103 patients (17.5%) were current tobacco users, which was higher than the proportion in the general population (11.7%). The overall smoking-attributable fraction of cancer deaths decreased significantly from 47.7% (95% CI, 47.3%-48.0%) in 2014 to 2016 to 44.8% (95% CI, 44.5%-45.1%) in 2017 to 2019, and this decrease was seen for both men and women. The overall smoking-attributable cancer mortality decreased by 10.2%.

**Conclusions and Relevance:**

California still has a substantial burden of tobacco use and associated cancer. The proportion of cancer deaths associated with tobacco use was almost double what was previously estimated. There was a modest but significant decline in this proportion for overall tobacco-associated cancers, especially for women.

## Key Points

**Question:**

What is the proportion and number of cancer deaths in California attributable to tobacco, using patient-level data?

**Findings:**

In this population-based cohort of 395 459 patients with a tobacco-associated cancer, nearly one-half of cancer deaths were associated with tobacco use, which is almost double what was previously estimated. Current tobacco use was higher than in the general population but decreased over time, leading to a modest but significant decline from 2014 to 2019 in the proportion of cancer deaths associated with tobacco.

**Meaning:**

These findings suggest that tobacco-associated mortality is high but modestly improving in California adults with a tobacco-associated cancer.

## Teaser

This cohort study evaluates the proportion and number of cancer deaths attributable to tobacco use using patient-level data in California.

## Introduction

Smoking is associated with 12 types of cancer (oral cavity and pharynx, larynx, esophagus, lung, liver, stomach, pancreas, kidney, urinary bladder, colon and rectum, uterine cervix, and acute myeloid leukemia [AML]) and remains the largest preventable factor associated with death from cancer and other diseases.^1^ The California Tobacco Control Program, established in 1989, has been associated with substantial decreases in cigarette consumption, lung cancer incidence, and heart disease mortality in California through supporting state and community interventions for policy, system, and environmental change on tobacco.^2,3,4^ California’s cigarette consumption has decreased faster compared with other states, and it now has the second lowest smoking rate in the US.^5^ From 1979 to 2005, California’s smoking-attributable cancer mortality (SACM) decreased by approximately 26% compared with 9% for the rest of the US, and from 1986 to 2013, its annual lung cancer mortality was 28% lower than in the rest of the US.^6,7^ Despite these successes, California still has more than 3 million people who smoke.^8^

Estimates from 2011 and 2014 of the proportion of cancer deaths attributable to smoking, the smoking-attributable fraction (SAF), found that smoking accounted for 26% of cancer deaths in California, compared with 49% in the US.^9,10^ However, these estimates relied on smoking prevalence data from general population surveys and did not use smoking data directly from patients with cancer. Furthermore, prior California SAF estimates were not specific to the 12 tobacco-associated cancers. Our study objectives were to determine more precise estimates of SAF and SACM for each of the tobacco-associated cancers in California using patient-level smoking information and to assess changes during 2014 to 2019. Such estimates can be used to further guide tobacco control efforts in California.

## Methods

### Data Source

The California Cancer Registry (CCR) is a state-mandated population-based cancer surveillance system that collects incidence reports on more than 180 000 new cases of cancer diagnosed annually in California. The CCR is composed of 3 National Cancer Institute Surveillance, Epidemiology, and End Results registries that collect data on tumor characteristics, patient demographics, and survival. Using CCR data, in this cohort study, we identified patients aged 20 years and older who received a diagnosis of 1 of the 12 tobacco-associated cancers using *International Classification of Diseases for Oncology, 3rd Edition* codes.^11^ We excluded 9588 patients whose cancers were diagnosed at autopsy or only through a death certificate. We used demographic characteristics defined by CCR to describe the cohort.

All analyses were overseen by the institutional review board of the University of California, Davis. Informed consent was waived because data were deidentified and all results presented in aggregate, in accordance with 45 CFR §46. This study is reported in accordance with the Strengthening the Reporting of Observational Studies in Epidemiology (STROBE) reporting guideline.^12^

### Tobacco Status Data

Current, former, and never tobacco status data collected by the CCR were from cancer tumor registrar documentation of patient information available at the time of diagnosis. The collected tobacco status data includes use of cigarettes, other smoked tobacco products (eg, cigars and pipes), and smokeless tobacco products (eg, chewing tobacco and snuff). The use of vaping products is not collected. For current and former smokers, we included data for other smoked tobacco products and smokeless tobacco (6.3% of tobacco use).

The CCR started collecting data in 2011 on documented tobacco status, which was required if available.^13^ There was a high level of missing tobacco status data in 2011 to 2013 for patients with a tobacco-associated cancer (142 192 patients [69.5%]). Because the missing level decreased over time, we focused on the 6 most recent diagnosis years (2014-2019) for which data were available and for which 37.4% of patients (147 873 patients; 75 609 patients [38.6%] for 2014-2016 and 72 264 patients [36.2%] for 2017-2019) were missing information on tobacco use. We compared patient demographic and clinical variables by missing tobacco status and found similar distributions for age group, neighborhood socioeconomic status (SES), stage, sex, marital status, comorbidity score, and cancer site other than lung. Slightly higher percentages of Hispanic patients and urban patients had missing tobacco status, whereas slightly lower percentage of non-Hispanic White patients, publicly insured patients, and patients with lung cancer had missing tobacco status (eTable 1 in Supplement 1). We imputed the missing tobacco status values using multiple imputation with the fully conditional method, which has been described elsewhere.^14^ We performed 20 imputations for each cancer site overall and for men and women separately. We used demographic variables as auxiliary variables in our imputation models, including age at diagnosis, race and ethnicity, SES, marital status, year of diagnosis, cancer stage at diagnosis, health insurance type, rural or urban residence, geographic area of the state, and Charlson Comorbidity Index score.^15^ We conducted sensitivity analyses excluding patients with missing tobacco information to compare with the imputed results. Race and ethnicity information were extracted from the CCR and were assessed in this study because smoking prevalence varies by race and ethnicity.^16,17^

### Statistical Analysis

We used descriptive statistics for describing demographic characteristics of the cohort and for analyzing tobacco status by cancer site and sex. We calculated quit ratios (percentage of ever users who have quit) by dividing the number of former users by the sum of current and former users. We calculated site-specific SAFs using the following standard formula^18^:
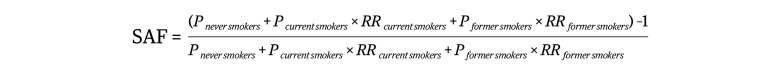
where *P_never smokers_* is the percentage of never smokers, *P_current smokers_* is the percentage of current smokers, *P_former smokers_* is the percentage of former smokers, *RR_current smokers_* is the relative risk of death for current smokers relative to never smokers, and *RR_former smokers_* is the relative risk of death for former smokers relative to never smokers. We used site-specific relative risks from large, pooled US analyses, including National Institutes of Health, American Association of Retired Persons, Diet and Health Study; Women’s Health Initiative, Cancer Prevention Study–II Nutrition Cohort; and the Nurses’ Health Study (eTable 2 in Supplement 1).^19,20,21^ We grouped diagnosis years into 2014 to 2016 and 2017 to 2019 and then calculated SAF by time period for each cancer site overall and for men and women separately. We used 2-sided χ^2^ tests to assess the equality of the SAF in the 2 time periods with a *P* < .05 level of significance.

We estimated the SACM for the 12 tobacco-associated cancers using methods similar to those used for the 2014 US Surgeon General’s Report.^1^ We identified the total number of cancer deaths for each cancer site by time period of patient diagnosis (2014-2016 and 2017-2019) using CCR data with follow-up limited to 1 year past the time periods, through 2017 and 2020 respectively, to have equivalent follow-up time for the 2 time periods. For the entire period, 2014 to 2019, we used all available follow-up time (through April 2022) to capture all cancer deaths. Next, we estimated the site-specific SACM by multiplying the total number of cancer deaths by each site’s SAF. Numbers of attributable deaths overall and by sex for each cancer type were calculated in separate estimation equations and rounded to the nearest whole number. Therefore, deaths by sex may not sum to the totals. Imputation and statistical analyses were done using SAS statistical software version 9.4 (SAS Institute).

## Results

Among 395 459 patients who had a tobacco-associated cancer, most (285 768 patients [72.3%]) were older than 60 years, the majority (228 054 patients [57.7%]) were non-Hispanic White, most were men (229 188 patients [58.0%]), more than one-half had private insurance (211 722 patients [53.5%]), and nearly one-half (184 415 patients [46.6%]) had lung or colorectal cancers (eTable 1 in Supplement 1). More than one-half ever used tobacco (227 660 patients [57.6%]), with 69 103 current users (17.5%) and 158 557 former users (40.1%) (Table 1). Approximately 1 in 5 men (44 987 patients [19.6%]) and 1 in 7 women (24 116 patients [14.5%]) with a tobacco-associated cancer were current tobacco users. More men were current tobacco users than women for each type of cancer, except for cancer of the larynx. Among men, the percentage of current tobacco users ranged from 30.2% (14 553 patients) for lung cancer to 10.9% (566 patients) for AML. Among women, the percentage of current tobacco users ranged from 37.5% (339 patients) for laryngeal cancer to 6.0% (247 patients) for AML. Tobacco use declined between time periods for all patients. An increase was seen in the proportion of never users (men, 37 616 patients [33.1%] to 42 067 patients [36.4%]; women, 42 229 patients [51.5%] to 45 887 patients [54.4%]) and ever users who have quit (quit ratio, men, 52 761 patients [69.3%] to 51 756 patients [70.5%]; women, 27 024 patients [67.9%] to 27 016 patients [70.4%]; overall quit ratio, 69.6% [158 557 patients]) over the 2 time periods.

**Table 1.** Tobacco Status Among Patients With a Newly Diagnosed Tobacco-Related Cancer by Site and Sex, California Cancer Registry, 2014-2019

Cancer site and tobacco use status	Patients, No. (%) (N = 395 459)					
	2014-2016		2017-2019		2014-2019	
	Male	Female	Male	Female	Male	Female
Acute myeloid leukemia						
Current	275 (11.1)	126 (6.5)	293 (10.9)	129 (6.0)	566 (10.9)	247 (6.0)
Former	986 (39.7)	515 (26.3)	1158 (43.0)	493 (22.9)	2132 (41.2)	1005 (24.4)
Never	1223 (49.2)	1316 (67.3)	1244 (46.1)	1535 (71.1)	2481 (47.9)	2862 (69.6)
Bladder						
Current	2771 (17.8)	735 (15.8)	2489 (16.0)	637 (13.7)	5250 (16.9)	1373 (14.7)
Former	7884 (50.6)	1683 (36.1)	7614 (49.0)	1704 (36.6)	15 542 (50.0)	3379 (36.3)
Never	4914 (31.6)	2244 (48.1)	5432 (35.0)	2312 (49.7)	10 313 (33.2)	4564 (49.0)
Cervix						
Current	NA	676 (15.5)	NA	574 (12.7)	NA	1252 (14.1)
Former	NA	843 (19.3)	NA	847 (18.8)	NA	1699 (19.1)
Never	NA	2847 (65.2)	NA	3091 (68.5)	NA	5929 (66.8)
Colorectal						
Current	3437 (14.9)	1871 (9.1)	3181 (13.7)	1698 (8.2)	6626 (14.3)	3570 (8.6)
Former	8519 (37.0)	4987 (24.1)	8268 (35.6)	4882 (23.5)	16 795 (36.3)	9881 (23.8)
Never	11 076 (48.1)	13 812 (66.8)	11 793 (50.7)	14 235 (68.4)	22 853 (49.4)	28 033 (67.6)
Esophagus						
Current	737 (21.9)	197 (18.4)	714 (19.7)	164 (15.9)	1452 (20.8)	354 (16.9)
Former	1745 (51.8)	465 (43.6)	1838 (50.8)	442 (42.9)	3590 (51.4)	909 (43.3)
Never	888 (26.3)	405 (38.0)	1065 (29.4)	425 (41.2)	1945 (27.8)	835 (39.8)
Kidney						
Current	1860 (16.0)	688 (10.6)	1735 (13.9)	634 (8.8)	3613 (15.0)	1336 (9.8)
Former	4538 (39.1)	1713 (26.4)	4702 (37.7)	1859 (25.9)	9242 (38.4)	3583 (26.2)
Never	5206 (44.9)	4097 (63.1)	6045 (48.4)	4695 (65.3)	11 230 (46.6)	8766 (64.1)
Larynx						
Current	697 (32.2)	175 (38.2)	584 (27.5)	162 (36.4)	1273 (29.7)	339 (37.5)
Former	1149 (53.1)	168 (36.8)	1125 (52.9)	192 (43.1)	2285 (53.3)	357 (39.5)
Never	316 (14.6)	115 (25.0)	417 (19.6)	92 (20.5)	731 (17.0)	208 (23.0)
Liver						
Current	1990 (22.6)	477 (13.3)	1858 (21.2)	440 (11.2)	3849 (21.9)	911 (12.2)
Former	4140 (47.0)	1017 (28.4)	3900 (44.5)	987 (25.2)	8047 (45.8)	2003 (26.8)
Never	2675 (30.4)	2081 (58.2)	3010 (34.3)	2487 (63.5)	5679 (32.3)	4574 (61.1)
Lung						
Current	7576 (31.0)	6296 (25.9)	6969 (29.3)	5460 (22.7)	14 553 (30.2)	11 776 (24.3)
Former	14 104 (57.7)	11 813 (48.6)	13 520 (56.8)	11 669 (48.4)	27 623 (57.2)	23 453 (48.5)
Never	2784 (11.4)	6194 (25.5)	3300 (13.9)	6972 (28.9)	6077 (12.6)	13 175 (27.2)
Oral						
Current	2051 (21.9)	598 (15.5)	1981 (20.6)	523 (13.3)	3997 (21.1)	1113 (14.3)
Former	4189 (44.8)	1274 (33.0)	4056 (42.3)	1201 (30.6)	8299 (43.8)	2488 (32.0)
Never	3114 (33.3)	1984 (51.5)	3557 (37.1)	2196 (56.0)	6652 (35.1)	4175 (53.7)
Pancreas						
Current	1142 (15.3)	684 (9.8)	1048 (12.9)	651 (8.5)	2197 (14.1)	1343 (9.2)
Former	3087 (41.5)	1830 (26.2)	3227 (39.8)	1976 (25.9)	6312 (40.6)	3793 (26.0)
Never	3211 (43.2)	4466 (64.0)	3832 (47.3)	5011 (65.6)	7037 (45.3)	9482 (64.9)
Stomach						
Current	804 (14.8)	225 (6.2)	795 (14.4)	296 (7.6)	1601 (14.6)	523 (7.0)
Former	2420 (44.5)	716 (19.8)	2348 (42.6)	764 (19.6)	4764 (43.5)	1482 (19.8)
Never	2209 (40.7)	2668 (73.9)	2372 (43.0)	2835 (72.8)	4583 (41.9)	5499 (73.3)
Total						
Current	23 340 (20.5)	12 748 (15.5)	21 647 (18.8)	11 368 (13.5)	44 987 (19.6)	24 116 (14.5)
Former	52 761 (46.4)	27 024 (33.0)	51 756 (44.8)	27 016 (32.1)	104 517 (45.7)	54 040 (32.5)
Never	37 616 (33.1)	42 229 (51.5)	42 067 (36.4)	45 887 (54.4)	79 683 (34.7)	88 116 (53.0)
Total 2014-2019	Current: 69 103 (17.5)		Former: 158 557 (40.1)		Never: 167 799 (42.4)	

Abbreviation: NA, not applicable.

The highest SAFs by type of cancer were for lung (60 316 patients [90.2%]), larynx (1726 patients [85.6%]), esophagus (3965 patients [58.0%]), oral cavity and pharynx (4932 patients [55.5%]), and bladder (7358 patients [52.7%]) cancers (Table 2). There were differences by sex in the SAF among the cancer sites. Men had higher SAFs than women for all cancer sites, except for cancers of the larynx and pancreas. The biggest differences in SAFs by approximately 20 percentage points between men and women were seen in liver cancer (4260 men [33.9%] vs 589 women [11.1%]), stomach cancer (1894 men [25.9%] vs 291 women [6.5%]), kidney cancer (1613 men [23.8%] vs 241 women [6.8%]), and AML (823 men [20.8%] vs 88 women [3.0%]).

**Table 2.** Number and Percentage of Cancer Deaths Attributable to Tobacco by Cancer Site and Sex, California, 2014-2019

Cancer site and year	Men		Women		Total	
	Total deaths, No.	Smoking attributable deaths, No. (%) [95% CI)	Total deaths, No.	Smoking attributable deaths, No. (%) [95% CI)	Total deaths, No.	Smoking attributable deaths, No. (%) [95% CI)
Acute myeloid leukemia						
2014-2016	1834	377 (20.6) [18.7-22.4]	1400	46 (3.3) [2.3-4.2]	3234	432 (13.4) [12.2-14.5]
2017-2019	1923	409 (21.3) [19.4-23.1]	1431	40 (2.8) [2.0-3.7]	3354	439 (13.1) [11.9-14.2]
2014-2019	3952	823 (20.8) [19.5-22.1]	2983	88 (3.0) [2.3-3.6]	6935	910 (13.1) [12.3-13.9]
Bladder						
2014-2016	4024	2215 (55.0) [53.5-56.6]	1397	672 (48.1) [45.5-50.7]	5421	2899 (53.5) [52.2-54.8]
2017-2019	4010	2145 (53.5) [51.8-55.2]	1333	621 (46.6) [43.9-49.3]	5343	2776 (51.9) [50.6-53.3]
2014-2019	10 584	5754 (54.4) [53.4-55.3]	3371	1595 (47.3) [45.6-49.0]	13 955	7358 (52.7) [51.9-53.6]
Cervix						
2014-2016	NA	NA	1109	117 (10.6) [8.8-12.4]	1109	117 (10.6) [8.8-12.4]
2017-2019	NA	NA	1035	95 (9.2) [7.4-11.0]	1035	95 (9.2) [7.4-11.0]
2014-2019	NA	NA	2594	257 (9.9) [8.7-11.1]	2594	257 (9.9) [8.7-11.1]
Colorectal						
2014-2016	6670	786 (11.8) [11.0-12.6]	5965	556 (9.3) [8.6-10.1]	12 635	1377 (10.9) [10.4-11.4]
2017-2019	6671	746 (11.2) [10.4-12.0]	5781	512 (8.9) [8.1-9.6]	12 452	1285 (10.3) [9.8-10.9]
2014-2019	17 099	1964 (11.5) [11.0-12.0]	14 690	1326 (9.0) [8.6-9.5]	31 789	3405 (10.7) [10.4-11.1]
Esophagus						
2014-2016	2364	1405 (59.4) [57.4-61.4]	733	411 (56.1) [52.5-59.7]	3097	1818 (58.7) [56.9-60.4]
2017-2019	2445	1419 (58.0) [56.0-60.1]	688	370 (53.8) [50.1-57.6]	3133	1798 (57.4) [55.6-59.1]
2014-2019	5267	3096 (58.8) [57.4-60.1]	1569	860 (54.8) [52.2-57.3]	6836	3965 (58.0) [56.8-59.2]
Kidney						
2014-2016	2683	656 (24.4) [22.8-26.1]	1419	99 (7.0) [5.7-8.3]	4102	710 (17.3) [16.1-18.5]
2017-2019	2598	599 (23.1) [21.3-24.8]	1427	93 (6.5) [5.2-7.8]	4025	650 (16.1) [15.0-17.3]
2014-2019	6783	1613 (23.8) [22.8-24.8]	3537	241 (6.8) [6.0-7.6]	10 320	1733 (16.8) [16.1-17.5]
Larynx						
2014-2016	668	555 (83.0) [80.2-85.9]	141	138 (97.7) [95.3-99.9]	809	699 (86.4) [84.0-88.8]
2017-2019	595	482 (81.1) [77.7-84.5]	143	140 (97.7) [95.2-99.9]	738	627 (85.0) [82.4-87.5]
2014-2019	1655	1358 (82.1) [80.2-83.9]	361	353 (97.7) [96.2-99.3]	2016	1726 (85.6) [84.1-87.2]
Liver						
2014-2016	5663	1959 (34.6) [33.4-35.8]	2329	275 (11.8) [10.5-13.1]	7992	2191 (27.4) [26.4-28.4]
2017-2019	5477	1821 (33.2) [31.9-34.5]	2461	251 (10.2) [9.0-11.4]	7938	2046 (25.8) [24.8-26.8]
2014-2019	12 552	4260 (33.9) [33.1-34.8]	5286	589 (11.1) [10.3-12.0]	17 838	4725 (26.5) [25.8-27.1]
Lung						
2014-2016	17 070	15 633 (91.6) [91.2-92.0]	14 857	13 292 (89.5) [89.0-90.0]	31 927	28 899 (90.5) [90.2-90.8]
2017-2019	15 274	13 936 (91.2) [90.8-91.7]	12 774	11 319 (88.6) [88.1-89.2]	28 048	25 232 (89.9) [89.6-90.3]
2014-2019	35 635	32 578 (91.4) [91.1-91.7]	31 200	27 784 (89.1) [88.7-89.4]	66 835	60 316 (90.2) [90.0-90.5]
Oral						
2014-2016	2552	1463 (57.3) [55.4-59.2]	1020	536 (52.6) [49.5-55.6]	3572	2018 (56.5) [54.9-58.1]
2017-2019	2514	1404 (55.8) [53.8-57.9]	989	489 (49.4) [46.3-52.6]	3503	1910 (54.5) [52.9-56.2]
2014-2019	6380	3604 (56.5) [55.2-57.7]	2500	1276 (51.0) [49.1-53.0]	8880	4932 (55.5) [54.5-56.6]
Pancreas						
2014-2016	5979	503 (8.4) [7.7-9.1]	5524	681 (12.3) [11.5-13.2]	11 503	1244 (10.8) [10.2-11.4]
2017-2019	6195	445 (7.2) [6.5-7.8]	5782	657 (11.4) [10.6-12.2]	11 977	1170 (9.8) [9.2-10.3]
2014-2019	12 876	1004 (7.8) [7.3-8.3]	11 966	1431 (12.0) [11.4-12.5]	24 842	2510 (10.1) [9.7-10.5]
Stomach						
2014-2016	3378	886 (26.2) [24.8-27.7]	2048	120 (5.9) [4.8-6.9]	5426	891 (16.4) [15.4-17.4]
2017-2019	3218	821 (25.5) [23.9-27.1]	2041	139 (6.8) [5.7-7.9]	5259	853 (16.2) [15.2-17.2]
2014-2019	7321	1894 (25.9) [24.9-26.9]	4463	291 (6.5) [5.8-7.2]	11 784	1927 (16.3) [15.7-17.0]
Total						
2014-2016	52 885	26 437 (49.9) [49.5-50.4]	37 942	16 943 (44.7) [44.2-45.2]	90 827	43 295 (47.7) [47.3-48.0]
2017-2019	50 920	24 228 (47.6) [47.2-48.1]	35 885	14 727 (41.0) [40.5-41.5]	86 805	38 880 (44.8) [44.5-45.1]
2014-2019	120 104	57 947 (48.2) [47.9-48.5]	84 520	36 090 (42.7) [42.3-43.0]	204 624	93 764 (45.8) [45.6-46.0]

Abbreviation: NA, not applicable.

A total of 204 624 deaths occurred among the cohort, with 93 764 smoking-attributable deaths (45.8%). Both the SACM and SAF overall decreased significantly over the 2 time periods (2014-2016 vs 2017-2019) and by sex; SACM decreased by 10.2%, from 43 295 deaths to 38 880 deaths, and the SAF decreased from 47.7% (95% CI, 47.3%-48.0%) to 44.8% (95% CI, 44.5%-45.1%) (Figure and Table 2). Among all patients in the cohort, the SAF decreased significantly for lung, liver, and pancreatic cancers. Among men, SACM decreased by 8.4%, from 26 437 deaths in 2014 to 2016 to 24 228 deaths in 2017 to 2019; the SAF overall decreased significantly from 49.9% (95% CI, 49.5%-50.4%) to 47.6% (95% CI, 47.2%-48.1%) between the same periods. By site, the SAF declined significantly only for pancreatic cancer in men. Among women, SACM decreased significantly by 13.1%, from 16 943 deaths in 2014 to 2016 to 14 727 deaths in 2017 to 2019; the SAF decreased from 44.7% (95% CI, 44.2%-45.2%) to 41.0% (95% CI, 40.5%-41.5%) between the same periods. For women, the SAF decreased significantly only for lung cancer.

**Figure.**
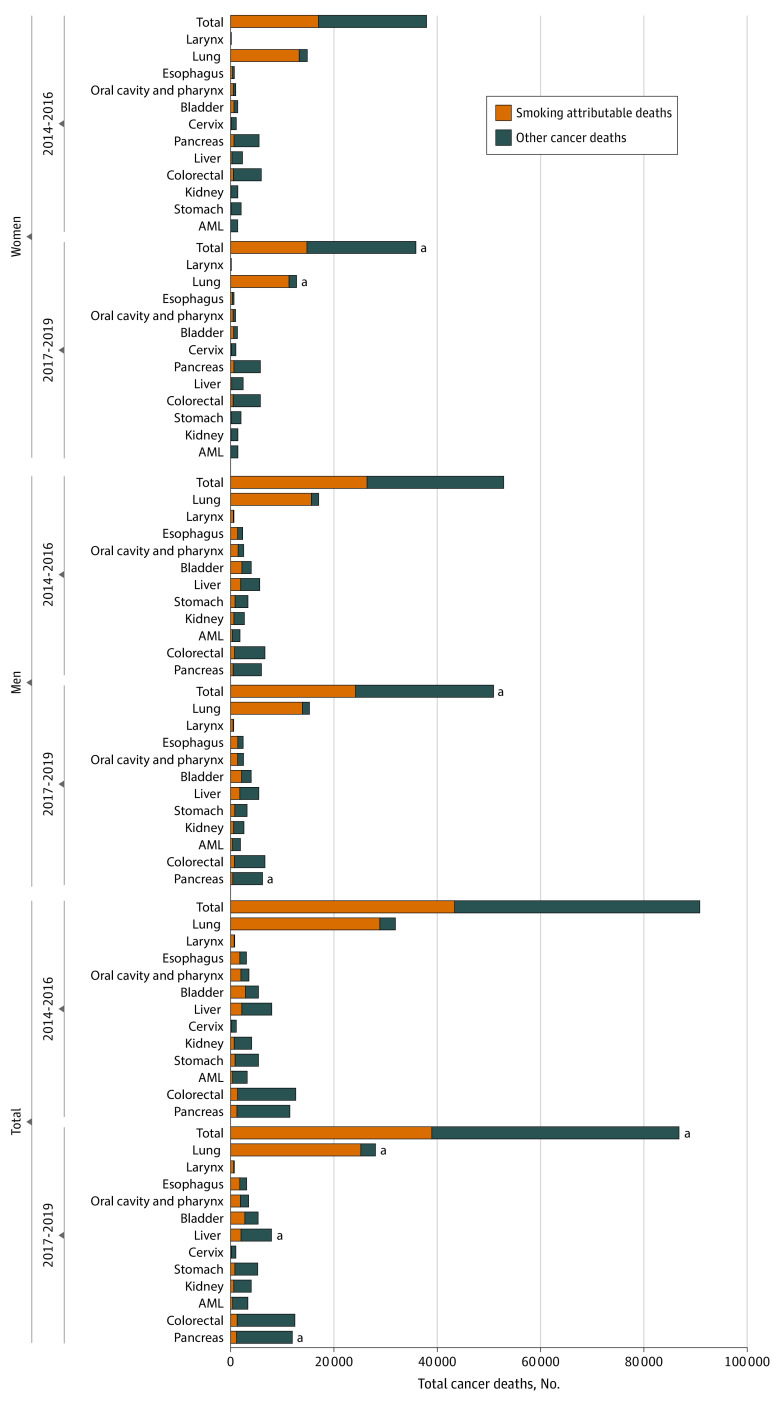
Cancer Deaths Associated With Tobacco Use Among California Men and Women by Year of Diagnosis Graph shows the number cancer deaths associated with tobacco use by cancer site and sex over 2 time periods. ^a^Significantly different from 2014 to 2016 at *P* < .05.

Among the 12 types of cancer, the top 5 SACM counts over the entire time period were for cancers of the lung (60 316 deaths), bladder (7358 deaths), oral cavity and pharynx (4932 deaths), liver (4725 deaths), and colon and rectum (3405 deaths). Among men, SACM was highest for cancers of the lung (32 578 deaths), bladder (5754 deaths), liver (4260 deaths), oral cavity and pharynx (3604 deaths), and esophagus (3096 deaths). For women, SACM was highest for cancers of the lung (27 784 deaths), bladder (1595 deaths), pancreas (1431 deaths), colon and rectum (1326 deaths), and oral cavity and pharynx (1276 deaths). For each cancer site, except for pancreatic cancer, men had greater SACM.

## Discussion

Despite California’s success in reducing tobacco use and decreasing cancer incidence and mortality,^22^ this cohort study found that there is a continued substantial burden of tobacco and mortality among patients with a tobacco-associated cancer in California from 2014 to 2019. Nearly one-half of the deaths (45.8%) in this cohort were attributable to tobacco, which reflects 93 764 Californians. This is almost double prior SAF estimates of 26% for California in 2014,^9^ for which smoking prevalence data were based on general population survey data. There was a modest but significant decrease in the SAF for overall tobacco-associated cancers, from 47.7% in 2014 to 2016 to 44.8% in 2017 to 2019. The greatest number of deaths attributable to tobacco for both men and women were from cancers of the lung and bladder.

Similar to a previous US study,^10^ we found differences in SACM and SAF between men and women. Although the SACM for overall tobacco-associated cancers decreased for both men and women, it decreased more for women. Of note, the SAF significantly decreased for women with lung cancer, which has a high SAF and contributes to the highest total of tobacco-associated cancers. The SAF also significantly decreased for men with pancreatic cancer, although this is a much lower total number. The wide differences in SAF between men and women for some cancers (liver, stomach, kidney, and AML) reflect differences in tobacco use in the cohort. Men largely had higher proportions of current tobacco use and lower proportions of never use than women and, thus, greater SAFs for most sites, except for laryngeal cancer, where women had higher proportions of current use and nearly identical proportions of never use in the most recent time period.

Our analyses of changes over the 2 time periods suggest promising trends in tobacco behavior among California patients with newly diagnosed cancer, which could be related to California’s ongoing tobacco control efforts. In November 2016, a $2 tobacco tax was passed by California voters that also helps support the activities of the California Tobacco Control Program, although there is a lack of evidence from survey estimates that smoking behavior in the general population continued to decrease significantly.^23^ However, the overall quit ratio in our study (69.6%) is a little higher than that for the general population in California (65.9%),^8^ and there was an increase in this ratio over the 2 periods, which reflects the periods before and after the implementation of the state tax. Additionally, the increasing proportion of never users over the 2 periods may reflect the growing proportion of never users since the California Tobacco Control Program started in 1989. However, the high percentage of current users in our cohort indicates that more smoking cessation efforts targeted at patients with cancer may be warranted. Quitting smoking after a cancer diagnosis can greatly reduce mortality and improve prognosis.^24^

### Strengths and Limitations

A major strength of our study was the tobacco status data derived from the patients with cancer in the study cohort, adding precision to our estimates. Prior studies^6,9,10,25^ used survey data about smoking prevalence in the general population, which likely underestimates the true proportion because of different smoking patterns between the general population and people who develop tobacco-associated cancers as our findings demonstrated. Ever tobacco use (57.6% vs 34.4%) and current tobacco use were higher (17.5% vs 11.7%) and the proportion of never smokers was much lower in the current study (men, 36.4%; women, 54.4%) than in the general population (men, 57.5%; women, 73.2%).^8^ Additionally, tobacco status data in the CCR reflected use of other smoked products (eg, cigars, pipes) and smokeless tobacco for a more comprehensive tobacco use measure.

Although patient-level data about tobacco are needed in cancer registries, accurate tobacco status data collection from electronic health records is challenging. First, there is not a standard question or definition of tobacco status in clinical practice. If a patient is asked, “Do you smoke now?” instead of about past-week use, it is possible to misclassify as many as 1 in 5 smokers eligible for lung cancer screening, or nearly 1.5 million people, as former smokers instead of current smokers.^26^ Most population surveys consider past-month use as a current tobacco user; however, population surveys may ask this question only if at least 100 cigarettes (or 5 packs) were smoked in the respondent’s lifetime.^26^ In a cross-sectional study^27^ of more than 16 000 current or former smokers eligible for lung cancer screening, more than 80% of evaluated records had inaccuracies in the tobacco history data (eg, missing years smoked, outdated data) in the electronic health records. Patient-reported data, such as with the National Cancer Institute’s Cancer-Tobacco Use Questionnaire,^28^ may be one way to collect more accurate data. The California Tobacco Control Program has been funding projects targeting health systems like CA Quits, which can help improve clinical workflow issues with tobacco screening and counseling.^29^

Cancer registries have potential for future research on tobacco and cancer. First, registry data may eventually have less missing data as more cancer programs regularly assess tobacco status. Nationwide, more than 700 cancer programs are working to improve the assessment and documentation of smoking status among patients newly diagnosed with cancer.^30^ Second, cancer programs are beginning to prioritize the integration of tobacco treatment in cancer care. The National Cancer Institute’s Cancer Center Cessation Initiative has funded cancer programs to implement tobacco treatment.^31^ Cancer registries could consider collecting future variables to measure tobacco treatment as part of cancer care, since quitting smoking improves cancer treatment outcomes and mortality as previously described.^24^

This study also has limitations that should be addressed. We had a high percentage of missing tobacco status (37.4%) but were able to impute the missing information using a validated method^14^ that incorporated patient-level variables. Sensitivity analyses excluding all patients with missing tobacco information yielded similar proportions of tobacco use (eTable 3 in Supplement 1) and SAFs to the main analyses with imputed data. However, SACM counts were much lower and likely underestimated because 37.4% of the cohort were excluded, illustrating that the imputed data set provides a better estimate of cancer deaths attributable to smoking.

Our estimates for SAF and SACM are based on smoking prevalence among California patients with cancer. It is possible that our findings may not be generalizable to the US population because California has the second lowest smoking rate in the country.^5^

We were unable to measure other tobacco exposures, such as vapes, which are increasing in use, especially in young people, or secondhand smoke exposure among nonsmokers, which has been classified as a known human carcinogen with no safe level of exposure,^32,33,34^ because the CCR does not collect this information. Furthermore, we could not assess the contribution of other substances that may be used with tobacco, such as marijuana or alcohol, which are known carcinogens, because data are also not routinely collected by the CCR. We acknowledge that some of the differences between our estimates and other estimates could be due to differences in methods and that direct comparisons may not be appropriate.

## Conclusions

The findings of this cohort study show that there is a higher tobacco-attributable burden of cancer in California than has previously been reported. Many patients with recent diagnoses of cancer are still current tobacco users, particularly those with cancers of the lung or larynx, of whom less than one-third still use tobacco. The modest but significant decrease in the SAF and SACM for overall tobacco-associated cancers suggests a population health benefit from California’s ongoing tobacco control efforts, but more research is needed to assess the outcomes of these efforts. Women with lung cancer may have benefited the most with the significant decline in SAF, but men with tobacco-associated cancers have the largest burden and need more attention for cessation. Cancer registries can be important data sources for monitoring progress in preventing tobacco-associated cancer incidence and mortality, implementing population-based tobacco control, and, in the future, also monitoring quality of cancer care with tobacco treatment to improve prognosis and reduce mortality. Future research can examine tobacco-associated cancer disparities for priority populations such as racial and ethnic minoritized groups, low SES populations, or geographic regions, as well as compare findings with cancer registries from the rest of the US.

## Notes

Supplement 1. **eTable 1.** Demographic Characteristics of Study Cohort **eTable 2.** Relative Risk of Death for Associations Between Smoking and Cancer Types **eTable 3.** Patient Tobacco Use Including Unknown Status (Not Imputed) by Cancer Site, 2014-2019, California
eReferences

Supplement 2.
Data Sharing Statement
