# Supplementary material for: Evaluation of Cancer Deaths Attributable to Tobacco in California, 2014-2019
Source: JAMA Netw Open. 2022 Dec 14;5(12):e2246651. doi: 10.1001/jamanetworkopen.2022.46651 (PMC9856507; doi:10.1001/jamanetworkopen.2022.46651)
Supplement: Supplement 1. — eTable 1. Demographic Characteristics of Study Cohort eTable 2. Relative Risk of Death for Associations Between Smoking and Cancer Types eTable 3. Patient Tobacco Use Including Unknown Status (Not Imputed) by Cancer Site, 2014-2019, California eReferences [file jamanetwopen-e2246651-s001.pdf]

# Supplemental Online Content

Maguire FB, Movsisyan AS, Morris CR, Parikh-Patel A, Keegan THM, Tong EK. Evaluation of cancer deaths attributable to tobacco in California, 2014-2019. *JAMA Netw Open*. 2022;5(12):e2246651. doi:10.1001/jamanetworkopen.2022.46651

**eTable 1.** Demographic Characteristics of Study Cohort

**eTable 2.** Relative Risk of Death for Associations Between Smoking and Cancer Types

**eTable 3.** Patient Tobacco Use Including Unknown Status (Not Imputed) by Cancer Site, 2014-2019, California

**eReferences**

This supplemental material has been provided by the authors to give readers additional information about their work.

© 2022 Maguire FB et al. *JAMA Network Open.*

## eTable 1. Demographic Characteristics of Study Cohort

| | Missing Tobacco | Not Missing Tobacco | Total |
|---|---|---|---|
| | n=147,873 | n=247,586 | n=395,459 |
| Characteristics | % (n) | % (n) | % (n) |
| Site | | | |
| AML | 2.3% ( 3466) | 2.4% ( 5827) | 2.3% ( 9293) |
| Bladder | 11.4% ( 16802) | 9.5% ( 23619) | 10.2% ( 40421) |
| Cervix | 2.1% ( 3076) | 2.3% ( 5803) | 2.2% ( 8879) |
| Colorectal | 23.3% ( 34445) | 21.5% ( 53313) | 22.2% ( 87758) |
| Esophagus | 2.1% ( 3073) | 2.4% ( 6012) | 2.3% ( 9085) |
| Kidney | 10.3% ( 15222) | 9.1% ( 22549) | 9.6% ( 37771) |
| Larynx | 1.2% ( 1846) | 1.4% ( 3346) | 1.3% ( 5192) |
| Liver | 6.6% ( 9778) | 6.2% ( 15285) | 6.3% ( 25063) |
| Lung | 21.8% ( 32215) | 26.0% ( 64442) | 24.4% ( 96657) |
| Oral Cavity & Pharynx | 7.0% ( 10287) | 6.6% ( 16437) | 6.8% ( 26724) |
| Pancreas | 7.2% ( 10597) | 7.9% ( 19567) | 7.6% ( 30164) |
| Stomach | 4.8% ( 7066) | 4.6% ( 11386) | 4.7% ( 18452) |
| Age group (years) | | | |
| 20-40 | 3.3% ( 4914) | 3.4% ( 8387) | 3.4% ( 13301) |
| 41-60 | 23.8% ( 35183) | 24.7% ( 61207) | 24.4% ( 96390) |
| 61-80 | 54.0% ( 79924) | 54.6% ( 135234) | 54.4% ( 215158) |
| >80 | 18.8% ( 27852) | 17.3% ( 42758) | 17.9% ( 70610) |
| Diagnosis year | | | |
| 2014 | 17.3% ( 25653) | 15.8% ( 39239) | 16.4% ( 64892) |
| 2015 | 17.1% ( 25215) | 16.2% ( 40102) | 16.5% ( 65317) |
| 2016 | 16.7% ( 24741) | 16.5% ( 40766) | 16.6% ( 65507) |
| 2017 | 16.9% ( 24990) | 16.7% ( 41407) | 16.8% ( 66397) |
| 2018 | 15.7% ( 23190) | 17.2% ( 42523) | 16.6% ( 65713) |
| 2019 | 16.3% ( 24084) | 17.6% ( 43549) | 17.1% ( 67633) |
| Race/ethnicity | | | |
| Asian/Pacific Islander | 14.0% ( 20776) | 13.0% ( 32106) | 13.4% ( 52882) |
| Hispanic | 23.3% ( 34464) | 19.0% ( 46939) | 20.6% ( 81403) |
| Native American | 0.6% ( 961) | 0.8% ( 1996) | 0.7% ( 2957) |
| NH Black | 7.4% ( 10886) | 6.0% ( 14792) | 6.5% ( 25678) |
| NH White | 52.5% ( 77663) | 60.7% ( 150391) | 57.7% ( 228054) |
| Other/Unknown | 2.1% ( 3123) | 0.6% ( 1362) | 1.1% ( 4485) |
| Neighborhood SES tertile | | | |
| Lowest | 30.9% ( 45671) | 29.2% ( 72401) | 29.9% ( 118072) |
| Middle | 37.3% ( 55134) | 37.6% ( 93069) | 37.5% ( 148203) |
| Highest | 31.8% ( 47068) | 33.2% ( 82116) | 32.7% ( 129184) |
| Charlson Comorbidity Score | | | |
| 0 | 28.4% ( 42038) | 30.6% ( 75643) | 29.8% ( 117681) |
| 1 | 18.0% ( 26629) | 20.5% ( 50823) | 19.6% ( 77452) |

© 2022 Maguire FB et al. *JAMA Network Open.*

| | | | |
|---|---|---|---|
| >1 | 29.0% ( 42860) | 29.8% ( 73809) | 29.5% ( 116669) |
| Unknown | 24.6% ( 36346) | 19.1% ( 47311) | 21.2% ( 83657) |
| AJCC Stage Group | | | |
| Stage I | 33.6% ( 49742) | 30.6% ( 75702) | 31.7% ( 125444) |
| Stage II | 11.7% ( 17297) | 13.5% ( 33330) | 12.8% ( 50627) |
| Stage III | 21.4% ( 31664) | 20.5% ( 50822) | 20.9% ( 82486) |
| Stage IV | 30.9% ( 45704) | 33.1% ( 81905) | 32.3% ( 127609) |
| Unstaged | 2.3% ( 3466) | 2.4% ( 5827) | 2.3% ( 9293) |
| Insurance | | | |
| Private/military | 53.4% ( 78951) | 53.6% ( 132771) | 53.5% ( 211722) |
| Public/Medicaid/Medicare | 38.7% ( 57173) | 43.9% ( 108718) | 41.9% ( 165891) |
| Uninsured | 0.8% ( 1177) | 1.0% ( 2490) | 0.9% ( 3667) |
| Unknown | 7.1% ( 10572) | 1.5% ( 3607) | 3.6% ( 14179) |
| Sex | | | |
| Female | 41.4% ( 61223) | 42.4% ( 105048) | 42.0% ( 166271) |
| Male | 58.6% ( 86650) | 57.6% ( 142538) | 58.0% ( 229188) |
| Marital Status | | | |
| Married | 51.3% ( 75838) | 53.3% ( 131972) | 52.5% ( 207810) |
| Not Married | 40.2% ( 59511) | 43.6% ( 107918) | 42.3% ( 167429) |
| Unknown | 8.5% ( 12524) | 3.1% ( 7696) | 5.1% ( 20220) |
| Residence | | | |
| Rural | 12.3% ( 18157) | 16.0% ( 39631) | 14.6% ( 57788) |
| Urban | 87.7% ( 129716) | 84.0% ( 207955) | 85.4% ( 337671) |
| Area | | | |
| Bay Area | 21.5% ( 31806) | 17.2% ( 42483) | 18.8% ( 74289) |
| Central | 25.8% ( 38212) | 22.4% ( 55362) | 23.7% ( 93574) |
| Northern | 9.3% ( 13692) | 21.8% ( 53978) | 17.1% ( 67670) |
| San Diego,Imperial,Orange | 14.4% ( 21289) | 18.1% ( 44749) | 16.7% ( 66038) |
| Los Angeles | 29.0% ( 42874) | 20.6% ( 51014) | 23.7% ( 93888) |

AML, acute myeloid leukemia; SES, socioeconomic status; AJCC, American Joint Committee on Cancer

© 2022 Maguire FB et al. *JAMA Network Open.*

## eTable 2. Relative Risk of Death for Associations Between Smoking and Cancer Types

**Males and Females**

| **Cancer type (ICD-0-3/WHO 2008)** | ***Smoking status (vs. never-smoking)*** | **Relative risk* (95% CI)** |
|---|---|---|
| AML (C92.0) | Current<br>Former | 1.57 (1.20–2.04)<br>1.30 (1.16–1.47) |
| Bladder (C67) | Current<br>Former | 3.90 (3.17–4.81)<br>2.37 (2.10–2.68) |
| Cervix (C53) | Current<br>Former | 1.59<br>1.14 |
| Colorectal (C18–C20, C26.0) | Current<br>Former | 1.51 (1.35–1.69)<br>1.20 (1.13–1.27) |
| Esophagus (C15) | Current<br>Former | 4.25 (3.44–5.24)<br>2.49 (2.18–2.85) |
| Kidney (C64–C65) | Current<br>Former | 1.55 (1.25–1.91)<br>1.38 (1.24–1.53) |
| Larynx (C32) | Current<br>Former | 17.4 (10.7–28.29)<br>2.78 (1.78–4.32) |
| Liver (C22.0, C22.1) | Current<br>Former | 2.10 (1.71–2.56)<br>1.38 (1.23–1.55) |
| Lung (C34) | Current<br>Former | 23.86 (22.31–25.51)<br>6.80 (6.39–7.24) |
| Oral cavity, pharynx (C00–C14) | Current<br>Former | 5.66 (4.34–7.38)<br>1.88 (1.53–2.31) |
| Pancreas (C25) | Current<br>Former | 1.74 (1.56–1.94)<br>1.08 (0.997–1.16) |
| Stomach (C16) | Current<br>Former | 1.81 (1.42–2.31)<br>1.30 (1.12–1.51) |

**Males**

| **Cancer type (ICD-0-3/WHO 2008)** | ***Smoking status (vs. never-smoking)*** | **Relative risk (95% CI)** |
|---|---|---|
| AML (C92.0) | Current<br>Former | 1.9 (1.4–2.7)<br>1.4 (1.2–1.6) |
| Bladder (C67) | Current<br>Former | 3.9 (3.0–5.1)<br>2.4 (2.1–2.8) |
| Colorectal (C18–C20, C26.0) | Current<br>Former | 1.4 (1.2–1.7)<br>1.2 (1.1–1.2) |
| Esophagus (C15) | Current<br>Former | 3.9 (3.0–5.0)<br>2.6 (2.2–3.0) |
| Kidney (C64–C65) | Current<br>Former | 1.8 (1.4–2.4)<br>1.5 (1.3–1.7) |
| Larynx (C32) | Current<br>Former | 13.9 (8.3–23.3)<br>2.4 (1.5–3.8) |
| Liver (C22.0, C22.1) | Current<br>Former | 2.3 (1.8–3.0)<br>1.5 (1.3–1.7) |
| Lung (C34) | Current<br>Former | 25.3 (22.8–28.1)<br>6.8 (6.2–7.5) |
| Oral cavity, pharynx (C00–C14) | Current<br>Former | 5.7 (4.1–8.1)<br>1.7 (1.3–2.2) |
| Pancreas (C25) | Current<br>Former | 1.6 (1.4–1.9)<br>1.0 (0.9–1.1) |
| Stomach (C16) | Current<br>Former | 1.9 (1.4–2.7)<br>1.5 (1.2–1.8) |

© 2022 Maguire FB et al. *JAMA Network Open.*

**Females**

| Cancer type (ICD-0-3/WHO 2008) | *Smoking status (vs. never-smoking)* | Relative risk (95% CI) |
|---|---|---|
| AML (C92.0) | Current<br>Former | 1.1 (0.7–1.7)<br>1.1 (0.9–1.4) |
| Bladder (C67) | Current<br>Former | 3.9 (2.8–5.5)<br>2.3 (1.8–2.9) |
| Cervix (C53) | Current<br>Former | 1.59<br>1.14 |
| Colorectal (C18–C20, C26.0) | Current<br>Former | 1.6 (1.4–1.9)<br>1.2 (1.1–1.3) |
| Esophagus (C15) | Current<br>Former | 5.1 (3.5–7.4)<br>2.2 (1.7–2.9) |
| Kidney (C64–C65) | Current<br>Former | 1.2 (0.9–1.8)<br>1.2 (1.0–1.4) |
| Larynx (C32) | Current<br>Former | 103.8 (24.2–445.5)<br>11.6 (2.7–49.6) |
| Liver (C22.0, C22.1) | Current<br>Former | 1.8 (1.3–2.5)<br>1.1 (0.9–1.4) |
| Lung (C34) | Current<br>Former | 22.9 (21.0–24.1)<br>6.8 (6.2–7.3) |
| Oral cavity, pharynx (C00–C14) | Current<br>Former | 5.6 (3.7–8.6)<br>2.2 (1.6–3.1) |
| Pancreas (C25) | Current<br>Former | 1.9 (1.6–2.2)<br>1.2 (1.1–1.4) |
| Stomach (C16) | Current<br>Former | 1.7 (1.2–2.5)<br>1.1 (0.9–1.4) |

*Relative risks come from large, pooled U.S. analyses[1-3]
AML, acute myeloid leukemia

© 2022 Maguire FB et al. *JAMA Network Open.*

## eTable 3. Patient Tobacco Use Including Unknown Status (Not Imputed) by Cancer Site, 2014-2019, California

| | | Total | Male | Female |
|---|---|---|---|---|
| Cancer Site | Smoking Status | n (%) | n (%) | n (%) |
| AML | Current | 538 (9.2%) | 369 (11.4%) | 169 (6.5%) |
| | Former | 1,946 (33.4%) | 1,307 (40.5%) | 639 (24.5%) |
| | Never | 3,343 (57.4%) | 1,548 (48.0%) | 1,795 (69.0%) |
| | *Unknown** | *3,466 (37.3%)* | *1,955 (37.7%)* | *1,511 (36.7%)* |
| Bladder | Current | 3,910 (16.6%) | 3,080 (17.0%) | 830 (15.0%) |
| | Former | 11,090 (47.0%) | 9,037 (50.0%) | 2,053 (37.1%) |
| | Never | 8,619 (36.5%) | 5,966 (33.0%) | 2,653 (47.9%) |
| | *Unknown** | *16,802 (41.6%)* | *13,022 (41.9%)* | *3,780 (40.6%)* |
| Cervix | Current | 858 (14.8%) | NA | 858 (14.8%) |
| | Former | 1,125 (19.4%) | NA | 1,125 (19.4%) |
| | Never | 3,820 (65.8%) | NA | 3,820 (65.8%) |
| | *Unknown** | *3,076 (34.6%)* | NA | *3,076 (34.6%)* |
| Colorectal | Current | 6,353 (11.9%) | 4,096 (14.6%) | 2,257 (8.9%) |
| | Former | 16,359 (30.7%) | 10,189 (36.4%) | 6,170 (24.4%) |
| | Never | 30,601 (57.4%) | 13,696 (48.9%) | 16,905 (66.7%) |
| | *Unknown** | *34,445 (39.2%)* | *18,292 (39.5%)* | *16,153 (38.9%)* |
| Esophagus | Current | 1,223 (20.3%) | 981 (21.2%) | 242 (17.4%) |
| | Former | 2,987 (49.7%) | 2,373 (51.3%) | 614 (44.2%) |
| | Never | 1,802 (30.0%) | 1,268 (27.4%) | 534 (38.4%) |
| | *Unknown** | *3,073 (33.8%)* | *2,365 (33.8%)* | *708 (33.7%)* |
| Kidney | Current | 3,010 (13.3%) | 2,192 (15.2%) | 818 (10.1%) |
| | Former | 7,708 (34.2%) | 5,541 (38.4%) | 2,167 (26.6%) |
| | Never | 11,831 (52.5%) | 6,680 (46.3%) | 5,151 (63.3%) |
| | *Unknown** | *15,222 (40.3%)* | *9,673 (40.2%)* | *5,549 (40.5%)* |
| Larynx | Current | 1,063 (31.8%) | 840 (30.5%) | 223 (37.6%) |
| | Former | 1,690 (50.5%) | 1,456 (52.9%) | 234 (39.5%) |
| | Never | 593 (17.7%) | 457 (16.6%) | 136 (22.9%) |
| | *Unknown** | *1,846 (35.6%)* | *1,535 (35.8%)* | *311 (34.4%)* |
| Liver | Current | 3,012 (19.7%) | 2,425 (22.6%) | 587 (12.9%) |
| | Former | 6,142 (40.2%) | 4,892 (45.6%) | 1,250 (27.4%) |
| | Never | 6,131 (40.1%) | 3,404 (31.8%) | 2,727 (59.8%) |
| | *Unknown** | *9,778 (39.0%)* | *6,854 (39.0%)* | *2,924 (39.0%)* |
| Lung | Current | 17,935 (27.8%) | 9,826 (30.8%) | 8,109 (24.9%) |
| | Former | 33,912 (52.6%) | 18,163 (56.9%) | 15,749 (48.4%) |
| | Never | 12,595 (19.5%) | 3,926 (12.3%) | 8,669 (26.7%) |
| | *Unknown** | *32,215 (33.3%)* | *16,338 (33.9%)* | *15,877 (32.8%)* |
| Oral Cavity and Pharynx | Current | 3,241 (19.7%) | 2,545 (21.6%) | 696 (14.9%) |
| | Former | 6,626 (40.3%) | 5,122 (43.5%) | 1,504 (32.2%) |
| | Never | 6,570 (40.0%) | 4,099 (34.8%) | 2,471 (52.9%) |
| | *Unknown** | *10,287 (38.5%)* | *7,182 (37.9%)* | *3,105 (39.9%)* |
| Pancreas | Current | 2,326 (11.9%) | 1,435 (14.1%) | 891 (9.5%) |
| | Former | 6,604 (33.8%) | 4,118 (40.5%) | 2,486 (26.5%) |
| | Never | 10,637 (54.4%) | 4,627 (45.5%) | 6,010 (64.0%) |
| | *Unknown** | *10,597 (35.1%)* | *5,366 (34.5%)* | *5,231 (35.8%)* |
| Stomach | Current | 1,347 (11.8%) | 1,028 (14.9%) | 319 (7.1%) |
| | Former | 3,912 (34.4%) | 2,995 (43.5%) | 917 (20.4%) |
| | Never | 6,127 (53.8%) | 2,857 (41.5%) | 3,270 (72.6%) |
| | *Unknown** | *7,066 (38.3%)* | *4,068 (37.2%)* | *2,998 (40.0%)* |
| Total | Current | 44,816 (18.1%) | 28,817 (20.2%) | 15,999 (15.2%) |

© 2022 Maguire FB et al. *JAMA Network Open.*

| | | | | |
|---|---|---|---|---|
| | Former | 100,101 (40.4%) | 65,193 (45.7%) | 34,908 (33.2%) |
| | Never | 102,669 (41.5%) | 48,528 (34.0%) | 54,141 (51.5%) |
| Total | *Unknown** | *147,873 (37.4%)* | *86,650 (37.8%)* | *61,223 (36.8%)* |

AML, acute myeloid leukemia

**eReferences**


1. Carter BD, Abnet CC, Feskanich D, et al. Smoking and mortality--beyond established causes. *The New England journal of medicine.* 2015;372(7):631-640.
2. Islami F, Goding Sauer A, Miller KD, et al. Proportion and number of cancer cases and deaths attributable to potentially modifiable risk factors in the United States. *CA: a cancer journal for clinicians.* 2018;68(1):31-54.
3. Centers for Disease Control and Prevention. Smoking-Attributable Mortality, Morbidity, and Economic Costs, Cancer Prevention Study II. https://academic.oup.com/view-large/19992493. Accessed October 13, 2022.


© 2022 Maguire FB et al. *JAMA Network Open.*
