# Supplementary material for: Evaluation of Cancer Deaths Attributable to Tobacco in California, 2014-2019
Source: JAMA Netw Open. 2022 Dec 14;5(12):e2246651. doi: 10.1001/jamanetworkopen.2022.46651 (PMC9856507; doi:10.1001/jamanetworkopen.2022.46651)
Supplement: Supplement 2. — Data Sharing Statement [file jamanetwopen-e2246651-s002.pdf]

Data Sharing Statement

Maguire. Evaluation of Cancer Deaths Attributable to Tobacco in California, 2014-2019. *JAMA Netw Open*. Published December 14, 2022. doi:10.1001/jamanetworkopen.2022.46651

Data
Data available: No

Additional Information
Explanation for why data not available: The data that support the findings of this study are available from the California Cancer Registry. Access is granted through an application process by the management or data custodians (https://www.ccrcal.org/retrieve-data/).
